# Enhancing photoluminescence yields in lead halide perovskites by photon recycling and light out-coupling

**DOI:** 10.1038/ncomms13941

**Published:** 2016-12-23

**Authors:** Johannes M. Richter, Mojtaba Abdi-Jalebi, Aditya Sadhanala, Maxim Tabachnyk, Jasmine P.H. Rivett, Luis M. Pazos-Outón, Karl C. Gödel, Michael Price, Felix Deschler, Richard H. Friend

**Affiliations:** 1 Cavendish Laboratory, Department of Physics, University of Cambridge, JJ Thomson Avenue, Cambridge CB3 0HE, UK

## Abstract

In lead halide perovskite solar cells, there is at least one recycling event of electron–hole pair to photon to electron–hole pair at open circuit under solar illumination. This can lead to a significant reduction in the external photoluminescence yield from the internal yield. Here we show that, for an internal yield of 70%, we measure external yields as low as 15% in planar films, where light out-coupling is inefficient, but observe values as high as 57% in films on textured substrates that enhance out-coupling. We analyse in detail how externally measured rate constants and photoluminescence efficiencies relate to internal recombination processes under photon recycling. For this, we study the photo-excited carrier dynamics and use a rate equation to relate radiative and non-radiative recombination events to measured photoluminescence efficiencies. We conclude that the use of textured active layers has the ability to improve power conversion efficiencies for both LEDs and solar cells.

## Abstract (web summary)

Recombinations govern losses in solar cells. Here, Richter *et al*. use transient spectroscopy to evaluate how re-absorption and re-emission of photons in perovskite absorbers affect intrinsic recombination coefficients, and to differentiate between external and internal photoluminescence quantum yields.

Organic–inorganic lead halide perovskites have emerged as disruptive materials for photovoltaics, with power conversion efficiencies recently exceeding 20% (ref. 1). Their exceptional performance has been attributed to efficient free charge generation^2^,^3^, long carrier lifetimes^4^, long excitation transport lengths^5^,^6^ and low apparent trap densities. Furthermore, the optical and electrical properties of lead halide perovskites can be tuned by their chemical composition. The optical absorption onset can be shifted across the visible to near-infrared region by changing the halide content from pure tri-iodide (band edge around 770 nm or 1.6 eV) to tri-bromide (band edge around 530 nm or 2.3 eV)^7^,^8^. The investigation of the semiconducting properties of this material class has been driven forward by spectroscopic measurements^9^,^10^,^11^. Transient photoluminescence (PL) experiments have been used to study the photo-physical and semiconducting properties of lead halide perovskites. It was found that the recombination processes of photo-excited charge carriers are strongly fluence-dependent^2^,^10^,^12^ and can give efficient radiative recombination from a bimolecular process. The recent demonstration of laser cooling^13^ and the excellent radiative efficiencies of nano-crystalline samples^14^ indicate the general possibility to achieve high radiative efficiencies in these materials. However, these findings are in conflict with reported, significantly lower external radiative efficiencies in thin film samples, which have been discussed in terms of non-radiative losses at defects and surfaces. This conflict raises the question, in how far externally measured radiative efficiencies give information on the internal recombination processes, or if these efficiencies are affected by other processes, such as light out-coupling, which is expected to be hindered by the relatively high refractive index of *n*∼2.7 (refs 15, 16). The understanding of photonic effects is crucial for the efficient operation of light-emitting devices^17^,^18^ and the ability to achieve high external efficiencies has been a crucial factor to push the power conversion efficiencies of single-junction photovoltaics^19^. Moreover, lead halide perovskites show sharp band edges and strong photoluminescence, which are the criteria required for photon recycling, that is, the re-absorption of radiatively recombining photo-generated charge pairs to regenerate an excitation. Our group has recently shown that photon recycling occurs in lead halide perovskites^20^. As developed below, this effect strongly affects externally observed recombination constants and, together with the high refractive index of these materials, leads to a significant difference between internally and externally measured photoluminescence quantum yields (PLQEs).

Here we investigate the recombination processes in lead halide perovskite thin films by quantitatively tracking photo-excited charge carrier densities with transient absorption (TA). In contrast to previous reports^12^,^21^,^22^, we can quantify the radiative fraction of the recombination rate by combining TA measurements with transient PL measurements. We find mono-, bi- and tri-molecular recombination regimes, of which only the bimolecular one is radiative. We show that photon recycling effects have to be taken into account to accurately determine intrinsic recombination coefficients, and demonstrate that internal PL quantum yields significantly exceed externally measured yields in thin film samples. By optimizing the light out-coupling with photonic structures, we recover the high internal efficiencies in external measurements, demonstrating the intrinsically efficient radiative recombination in lead halide perovskites.

## Results

### Enhancing photoluminescence yields by light out-coupling

**Figure 1:**
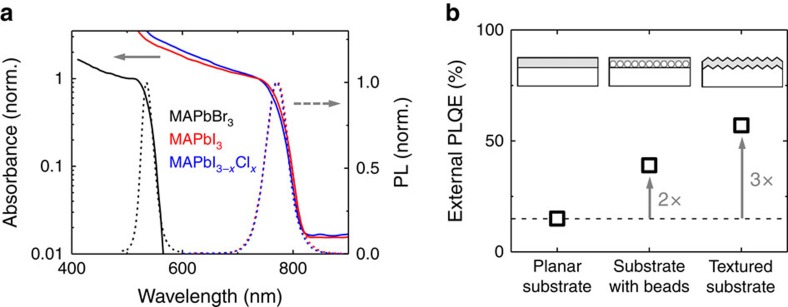
Radiative efficiency of perovskite films. (**a**) Steady-state absorption spectra measured with photothermal deflection spectroscopy (solid lines) and photoluminescence spectra (dashed lines) for MAPbX_3_ (X=I, Br, I_1−*x*_Cl_*x*_). (**b**) External PLQEs for MAPbI_3−*x*_Cl_*x*_ on different substrates. The film on a textured substrate shows an external PLQE of 57%, three times higher than the planar film with 20% (indicated by dashed line).

We prepared thin films of CH_3_NH_3_PbI_3_, CH_3_NH_3_PbI_3-*x*_Cl_*x*_ (that is, PbCl_2_ used in preparation) and CH_3_NH_3_PbBr_3_ on glass substrates. All films were deposited in a single step spin coating process of a precursor solution based on methylammonium halide mixed with lead acetate or lead chloride in DMF. Absorption and PL emission spectra of the films can be seen in Fig. 1a. All films show long-term photostability under the investigated fluences for both pulsed and continuous wave excitation. We did not observe a light soaking effect which is an increase in luminescence intensity over minutes of illumination^23^ but reach high PLQEs within the turn-on time of the laser. We measure the external PLQEs of thin films of these three material compositions under continuous wave (CW) laser excitation at 532 nm (407 nm for bromide perovskite) and measure 5%, 20% and 15% for CH_3_NH_3_PbI_3_, CH_3_NH_3_PbI_3−*x*_Cl_*x*_ and CH_3_NH_3_PbBr_3_, respectively, comparable to literature values of perovskite thin films^24^,^25^. To investigate the importance of light out-coupling and photon recycling on the externally measured PLQEs, we change the planar sample structure by depositing perovskite films on a textured glass substrate with structures on the length scale of 100 nm to 1 μm (for characterization see Supplementary Figs 1 and 2). In these samples, for the iodide–chloride perovskite, we measure an external PLQE of 57% in the structured film, compared with 20% in the planar reference film, which was made from the same precursor solution on an unstructured substrate. For the iodide perovskite, the external PLQE increases by a factor of 5 from 5 to 27%. In addition, we deposited silicon dioxide microspheres (1 μm diameter, Sigma Aldrich) on the glass substrate before spin coating the perovskite film on top of these ‘microlenses' that leads to a rise of the external PLQE for the iodide–chloride film to 39% and for the iodide film to 17%. Figure 1b summarizes the PLQEs for iodide–chloride perovskite on different substrates. This demonstrates that light out-coupling determines the external luminescence intensity of lead halide perovskites.

### Time-resolved spectroscopy identifies recombination mechanisms

To further understand how recombination and photon recycling affects the external PLQEs, we study the time-resolved carrier dynamics in lead halide perovskites by performing transient absorption (TA) and transient photoluminescence (PL) measurements on planar films of all three material compositions. To minimize carrier diffusion effects^5^ on the measured recombination processes, we chose excitation spot sizes larger than 200 μm.

**Figure 2:**
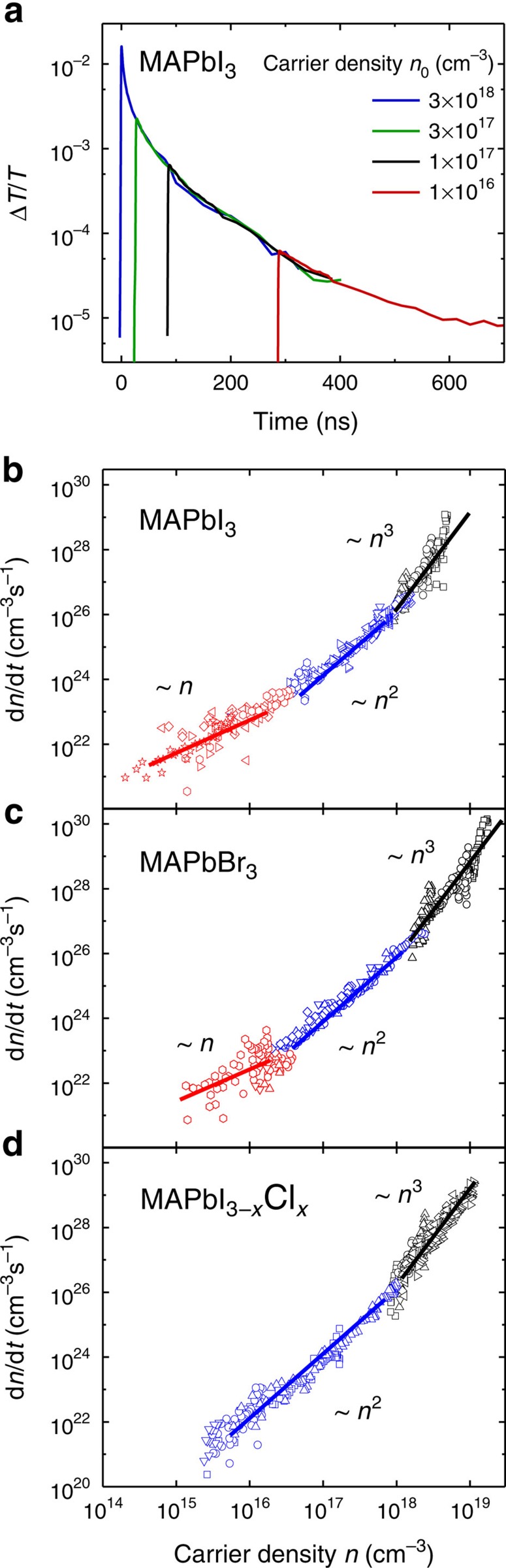
Carrier density dynamics of perovskites. (**a**) TA kinetics of MAPbI_3_ after 650 nm 100 fs pulsed excitation. Time zero for different fluences was shifted to overlay absolute values with the respective next higher fluence. We find that the transient decays match very well and conclude that the recombination rate d*n/*d*t* is only dependent on carrier density *n*(*t*). (**b**–**d**) Recombination rate d*n/*d*t* over charge density *n* for (**b**) MAPbI_3_, (**c**) MAPbBr_3_ and (**d**) MAPbI_3−*x*_Cl_*x*_. The values were extracted from TA measurements for ∼10 different fluences with each symbol representing one measurement. The normalized TA kinetics were multiplied with the initial charge carrier density *n*_0_ (determined from fluence and absorption values, see Supplementary Note 1) and taking the time-derivative of this carrier density *n*(*t*). We observe different scalings of the recombination rate d*n/*d*t* with *n* indicating transitions between different recombination mechanisms. The solid lines are a guidance to the eye.

Typical TA spectra can be found in Supplementary Fig. 4a that have been described in detail elsewhere^9^,^26^,^27^. We spectrally integrate over the ground state bleach of the TA spectra (in a range of 100 meV over the peak at 1.65 eV for iodide and at 2.35 eV for bromide perovskite) to get a measure that is proportional to the excitation density *n*(*t*). We confirm that this is a good measure for the carrier density by showing that the initial TA signal after excitation is linear in pump power (Supplementary Fig. 4b). The initial carrier density *n*_0_ is estimated from the absorption and thickness of the films (see Supplementary Note 1). Figure 2a shows the TA kinetics of iodide perovskite after pulsed laser excitation for different fluences. For this graph, the time zero of individual measurements was shifted along the time axis to match the respective next higher carrier density. We find that the TA signal decays smoothly over multiple orders of magnitude. From this, we conclude that the recombination rate 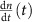 only depends on the excitation density *n*(*t*) and is ‘history-independent'^28^. We find the same result for bromide and iodide–chloride based methylammonium lead halide perovskites and in PL measurements (Supplementary Fig. 3).

We multiply the TA kinetics with the initial carrier density *n*_0_ to get a measure for *n*(*t*) (see Supplementary Note 1 for estimate of *n*_0_). By taking the time-derivative of the carrier density 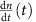, and plotting it over the carrier density *n*(*t*), we derive carrier density-dependent recombination rates that can be found in Fig. 2b–d for all three material compositions. We find that the recombination rates are higher for the iodide film compared to the iodide–chloride and bromide film. For all three compositions, we find different scaling regimes of the recombination rate with carrier density *n*. At low carrier densities (*n*<10^16^ cm^−3^) the rate is linear in carrier density for iodide and bromide perovskite, while at higher carrier densities we find a quadratic (*n* =10^16^–10^18^ cm^−3^) and cubic (*n*>10^18^ cm^−3^) dependence. The monomolecular regime was not resolved in the TA experiment for the iodide–chloride sample, but was clearly present in low-fluence transient PL measurements (Supplementary Fig. 5). We conclude that mono-, bi- and trimolecular charge carrier recombination pathways are present in all three perovskite materials and can describe the recombination rates with the general rate equation


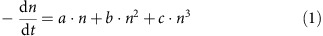


By fitting the recombination rates with this rate equation, we find the recombination constants reported in Table 1. We compare the extracted monomolecular constants with the constants from low-fluence transient PL measurements (Supplementary Fig. 5) and find a good agreement.

To distinguish between radiative and non-radiative recombination pathways, we perform transient PL measurements. Transient photoluminescence is a measure for the radiative part of the recombination rate


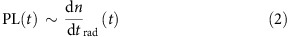


**Figure 3:**
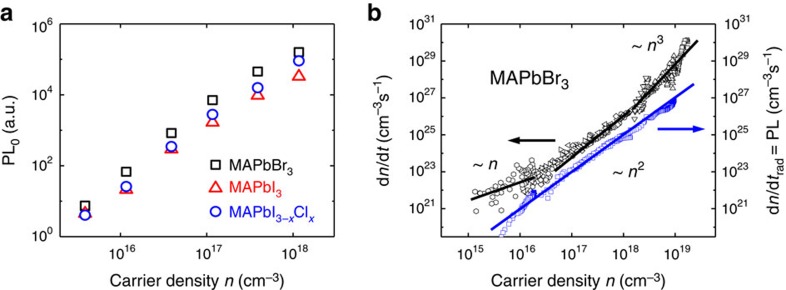
Radiative recombination in perovskites. (**a**) Initial transient PL signal (time integrated for 2 ns after excitation with a 100 fs laser pulse) plotted over the initial charge carrier density. We observe a quadratic scaling in *n* in all three material compositions. (**b**) Total (black) and radiative (blue) recombination rate dependence on charge carrier density *n*. The radiative recombination rates were derived by plotting the transient PL signal over the TA signal at the same time delay. The radiative recombination rate scales quadratically with carrier density even in regimes where the total recombination rate is linear (below 2 × 10^16^ cm^−3^) and cubic (above 2 × 10^18^ cm^−3^) in excitation density. We conclude that the radiative recombination is bimolecular and that linear and cubic recombination rates have their origin in non-radiative processes.

Figure 3a shows the setup-limited (time resolution about 1.5 ns), initial PL signal, PL_0_, for pulsed optical excitation. We find that the initial PL signal scales quadratically with pump power and thus carrier density for all three material compositions. In addition, we plot 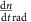, which is determined from the transient PL signal, over the TA signal for the same time delay, that is, with time as the intrinsic variable, as seen in Fig. 3b for MAPbBr_3_ (other materials in Supplementary Fig. 6). We note that the PL is proportional to the radiative recombination rate and TA is proportional to the carrier density. We observe that the radiative recombination rate scales quadratically with carrier density and has therefore its origin in a bimolecular process for all investigated charge carrier densities. We attribute this to a direct band-to-band transition with a constant bimolecular recombination constant *b*_rad,ext_ in the investigated fluence regime. This has been shown before for iodide perovskite^2^,^29^ but is still debated for bromide perovskite where the role of the exciton has to be clarified. We find that the dominant radiative recombination channel in bromide perovskite is a band-to-band recombination rather than an excitonic monomolecular recombination at the investigated fluences. Interestingly, the radiative rate 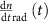 still scales quadratically with *n* in the lower fluence regime, where the total charge carrier recombination rate 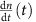 is dominated by the monomolecular regime. We therefore conclude that the origin of the monomolecular recombination is a non-radiative process, while the radiative recombination is still bimolecular at these lower fluences (*n*<10^16^ cm^−3^). We did not find evidence for a radiative monomolecular process in any of the investigated material compositions over the investigated fluence regime. This indicates that the properties of the excited states in lead halide perovskite are dominated by free charge pair behaviour rather than bound excitons.

### Impact of photon recycling on external PLQEs

**Figure 4:**
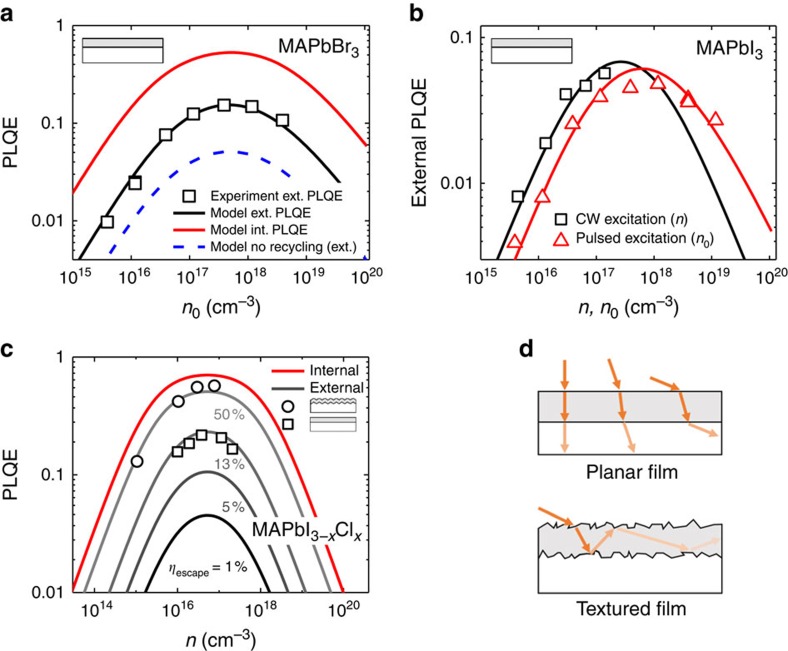
Fluence dependence of perovskite PLQEs. (**a**) External PLQEs for MAPbBr_3_ under pulsed laser excitation (squares) together with the modelled internal (red) and external (black) PLQEs from equation (6). The internal PLQEs reach values up to 70%. The blue line represents the model without photon recycling. (**b**) Measured external PLQEs for MAPbI_3_ under steady state (black) and pulsed laser excitation (red) with the prediction from the recombination rate model (lines). Under pulsed excitation, the averaged PLQE is measured over the decay of the carrier density *n*. (**c**) Measured external PLQEs of MAPbI_3−*x*_Cl_*x*_ films when deposited on a textured (circles) and planar (squares) substrate under CW excitation, together with computed PLQEs for different photon escape probabilities *η*_esc_. While we were estimating the escape probability for the planar film to be 12.7%, we determine an escape probability of about 50% for the textured substrate, yielding an external PLQE of 57%. (**d**) Sketch illustrating the improved sunlight in-coupling of textured solar cells. In a planar film, light will leave the film at the second interface. In a textured film, light undergoes about 30 total internal reflections and can therefore travel significantly longer in the active layer.

To understand the impact of the different recombination pathways on externally measured PL quantum efficiencies, we perform fluence-dependent PLQE measurements under pulsed and CW laser excitation. Figure 4a shows the measured external PLQEs for bromide perovskite under pulsed excitation (squares). The PLQE shows a rise at low fluences, peaks around 3 × 10^17^ cm^−3^ and then falls at higher fluences. To understand the fluence dependence of the PLQE, we have to consider the fluence-dependent recombination rates, as well as the effects of photon recycling.

It has been shown in seminal studies of inorganic semiconductors^30^,^31^, that emitted PL photons can be re-absorbed and re-emitted if the photoluminescence and absorption spectra overlap. Such an effect has been recently shown in lead halide perovskites^20^. The effect of this filtering and re-emission can be substantial if PL photons are trapped in the active layer due to total internal reflection. Perovskite has a high refractive index of *n*≅2.7 at the band edge^16^ leading to an escape cone for emitted photons at the perovskite-air interface of only *α*=sin^−1^(*n*)=22°. Above this critical angle, photons will be trapped in the film and eventually be re-absorbed. By considering the perovskite-glass and perovskite-air interfaces, we estimate the escape probability, which is the probability that an emitted photon will leave the film before re-absorption, to be *η*_esc_=12.7% for the planar film of iodide perovskite (details in Supplementary Note 2). This is an upper boundary for the escape probability as discussed in Supplementary Note 2 providing a lower boundary (and thus conservative estimate) for the internal PLQE later. We can then derive an expression for the external PLQE *η*_ext_ by writing it as a series over multiple re-absorption events^31^,^32^,^33^:


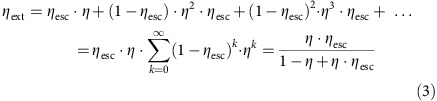


where *η* is the internal PLQE. Furthermore, the recycling of photons will affect the radiative recombination constant *b*_rad_. The intrinsic recombination constant *b*_rad,int_ will be higher than the externally measured *b*_rad,ext_ due to a slowing down of the carrier concentration decay by re-absorption of photons. We can derive an expression between the two by noting that the change in carrier density *n* we observe must be the same internally and externally, which leads to


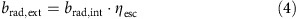


The non-radiative recombination constants will be unaffected by photon recycling, because non-radiatively recombining charges cannot be subsequently recycled.

We expect the fluence dependent PLQE to be described by / radiative and total recombination rates according to


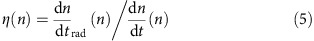


If we assume that only the bimolecular process is radiative, we expect


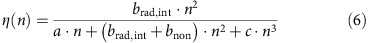


for the internal PLQE. We assume that we have non-radiative mono-, bi- and trimolecular recombination (with recombination constants *a*, *b*_non_ and *c*, respectively) competing with the radiative bimolecular process (with bimolecular constant *b*_rad,int_). Under photon recycling, the external PLQE can be calculated using equation (3) or by using equation (6) with the external recombination constant *b*_rad,ext_ instead of *b*_rad,int_ for the radiative recombination.

Using the recombination constants listed in Table 1, we plot the computed external PLQE according to equation (6) together with the experimental PLQE data in Supplementary Fig. 7a assuming that the bimolecular recombination is purely radiative, corresponding to *b*_non_=0. We observe that there is a significant discrepancy between this model and the experimentally observed PLQEs. We note that this discrepancy is independent of the estimate for the escape probability, since we have only used the externally measured recombination constants, and is not accounted for by imperfect light trapping. We conclude that the bimolecular recombination constant is not purely radiative but has a non-radiative component. This also explains the offset of the total recombination rate to the radiative recombination rate in the bimolecular regime in Fig. 3b (Supplementary Fig. 6 for iodide and iodide–chloride perovskite). A known non-radiative bimolecular process is for example trap-assisted Auger recombination^34^. Another model picture is that the carriers are spending most of their time in traps but are still diffusing between them. The recombination kinetics could then look bimolecular, but some carriers would recombine via traps. The origin of this recombination pathway, however, has still to be clarified. Figure 4a shows the external PLQE for bromide perovskite. The blue dashed line represents the model assuming no photon recycling that under-estimates the external PLQEs. This re-confirms that photon recycling is happening in these perovskite structures. By fitting equation (6) to the experimental PLQE data with *b*_non_ as the only fitting parameter, we derive radiative and non-radiative bimolecular recombination constants as reported in Table 2. The black line in Fig. 4a represents the fit according to this model and the red line shows the calculated internal PLQE according to equation (3). We conclude that internal PLQEs in perovskite can exceed 70%. This is, however, an estimate as there is no direct way to measure internal PLQEs for these films due to the non-linearity of internal and external PLQE (equation (3)).

Figure 4b shows measured external PLQEs for MAPbI_3_ under pulsed and CW excitation. Under pulsed excitation, we can reach high excitation densities, which proves difficult with CW excitation due to the strong increase in recombination rates as seen in Fig. 2. When measuring PLQEs under pulsed excitation, we observe a time average of the PLQE for different carrier densities during the transient PL decay. The continuous lines represent the model discussed before, where the PLQE under pulsed excitation is the CW PLQE averaged over *n* from 0 to *n*_0_. We find that the measured PLQEs under pulsed and CW excitation are in good agreement with this model.

Figure 4c shows calculations of internal and external PLQEs according to equations (3) and (6) for different escape probabilities *η*_esc_ together with the experimental PLQE for the iodide–chloride film on the textured and planar substrate. By structuring the substrate, we have increased the escape probability from 7% to above 50%.

## Discussion

The dominant PL emitting process is bimolecular. The radiative emission rate of perovskites is therefore not constant but linear in excitation density. We find that the reported monomolecular lifetimes at low fluences^5^,^21^,^35^ have their origin in a non-radiative recombination channel. The light-emitting process, however, is still bimolecular. This implies that the decays measured in transient PL measurements are twice as fast as the actual carrier population decay. One therefore has to multiply the monomolecular lifetime measured in low fluence PL experiments by 2 in order to get the actual carrier lifetime.


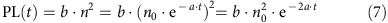


We attribute this monomolecular non-radiative recombination to a trap-assisted Shockley-Read-Hall recombination, which has been reported before and studied in detail^25^,^36^,^37^. The processes of carrier trapping, de-trapping and subsequent recombination can be described in more detail, but this is beyond the scope of this work. We attribute the radiative bimolecular recombination process to a band-to-band transition and the three-particle recombination to an Auger process. When comparing the pure iodide with the iodide–chloride perovskite, we observe that their bimolecular and Auger constants are very similar while the *a* constant is almost an order of magnitude higher for the pure iodide film than for the chloride-doped material. The lower monomolecular recombination in chloride-doped perovskite points towards a lower trap density in these films, which will lead to much more efficient photon recycling and thus higher power conversion efficiencies in photovoltaics. The main difference in carrier recombination in these two material compositions is therefore the formation of fewer defect sites in chloride-doped films, rather than an intrinsic difference in the carrier–carrier interaction.

Photon recycling can boost external PLQEs significantly as shown in Fig. 4a. With an escape probability of ∼12.5%, 7 out of 8 emitted photons will stay in the perovskite film and get re-absorbed. For our iodide–chloride planar films, we measured 20% external PLQE. Without re-emission of the re-absorbed photons, this number would have been as low as *η*·*η*_esc_=70%·12.7%=8.9%.

Photon recycling does affect the apparent radiative bimolecular constant. The relation between these two is given by the escape probability for photons, see Table 2. The internal recombination constant can be eight times higher than the externally observed constant.

The direction of luminescence emission plays an important role and affects the escape probability. We performed all measurements on pure perovskite films on glass substrates. We therefore had to distinguish between the two interfaces perovskite-glass and perovskite-air. The introduction of rear mirrors simulates the situation of an optoelectronic device where the rear electrode often acts like a mirror, and has been studied in detail^30^. It has been shown that in such samples the external PLQE strongly depends on the quality of the mirror due to parasitic absorption losses. Studying the pure perovskite film on glass is a best-case scenario for emission as our samples do not contain any additional layers that could cause parasitic absorption of luminescence. In a device, any additional layers could cause a small proportion of parasitic absorption that would affect the external luminescence intensity.

Efficient photon recycling can be highly beneficial for power conversion efficiencies of solar cells. The emission of luminescence under open circuit will give an additional photon field to the sunlight radiation leading to an overall higher photon density in the semiconductor compared with the case without photon recycling. This effect has been reported to produce higher open-circuit voltages^30^,^38^. For significant boosts in open-circuit voltage, a very high external PLQE under open-circuit condition is necessary. The dependence of the open-circuit voltage has been found to be^30^,^39^,^40^


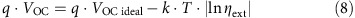


where *V*_OC ideal_ denotes the open-circuit voltage in the radiative limit, that is, under unity external PLQE^40^. An increase in external PLQE at solar fluences (excitation density of 10^15^ cm^−3^) from 1% to close to unity would therefore bring a voltage increase of 0.12 V. The external PLQE *η*_ext_ has been found to depend both on internal PLQE and the light escape probability according to equation (3). The external PLQEs can thus be enhanced by increasing the internal PLQEs and by improving the light out-coupling via surface roughening. For solar cells, a performance boost can be achieved by either an efficient photon recycling process that requires very high internal PLQEs, causing a voltage gain, or by randomising the surface of the film. In the latter case, the PL out-coupling under open-circuit condition is improved as well as the in-coupling of sunlight. This leads to higher short-circuit currents and to power conversion efficiencies exceeding those of planar films as shown for GaAs by Miller *et al*.^30^ For internal PLQEs lower than 95% and the corresponding external PLQEs lower than 50% (according to equation (3)), photon recycling becomes detrimental due to the non-radiative losses in each recycling step. The textured solar cell design is then particularly favourable. The surface of the textured film randomizes the angle of the incoming sunlight light as it enters the perovskite film. The ‘trapped' photons will then have a small escape probability of 1/4*n*^2^=1/30 at each bounce. Light will therefore travel up to 30-times further in the active layer (this value is slightly reduced in sub-wavelength thick active layers^41^) than in the planar film as illustrated in Fig. 4b. This allows to make much thinner active layers compared with the planar structure without compromising photon absorption. This increases the concentration of charges in the perovskite layer, reduces the series resistance of the device and improves the fill factor.

Solar cells and LEDs are reciprocal devices. Thus, for light-emitting applications, the structure of the device and surfaces will have strong influence on the external luminescence. Light trapping reduces the external light intensity in planar film LEDs. Approaches similar to those demonstrated here can be used to increase the light out-coupling of perovskite LEDs and therefore significantly increase the external quantum efficiencies. This includes a texturing of the substrate or of the film surface, the use of microlenses to suppress total internal reflection or a refractive index matching of the substrate.

## Methods

### Film preparation

For the iodide and bromide perovskite films: 3:1 molar stoichiometric ratios of CH_3_NH_3_I and CH_3_NH_3_Br for bromide and Pb(CH_3_COO)_2_ (Sigma Aldrich 99.999% pure) were made in *N*,*N*-dimethylformamide (DMF) in 20wt% solution. These solutions were spun inside a nitrogen filled glove box on quartz substrates at 2,000 r.p.m. for 60 s followed by 3 min of thermal annealing at 100 °C in air to form thin films. For iodide–chloride perovskite, PbCl_2_ was used in the precursor instead of Pb(CH_3_COO)_2_.

### Preparing the glass substrates

Perovskite films were deposited on planar glass substrates, glass substrates with beads and rough glass substrates. For the substrate with beads, a solution of silica beads with 1 μm diameter (Sigma Aldrich microparticles, 56798-5ML-F) was spin coated on top of a flat glass substrate. For the rough substrates, a flat glass substrate was abraded with sandpaper with 1,200 grains per inch giving structures on the length scale of 100 nm to 1 μm.

### Photothermal deflection spectroscopy

Photothermal deflection spectroscopy (PDS) is a highly sensitive surface averaged absorption measurement technique. For the measurements, a monochromatic pump light beam produced by a combination of a Light Support MKII 100W Xenon arc source and a CVI DK240 monochromator, is shined on the sample (film on Quartz substrate), inclined perpendicular to the plane of the sample, which on absorption produces a thermal gradient near the sample surface via non-radiative relaxation induced heating. This results in a refractive index gradient in the area surrounding the sample surface. This refractive index gradient is further enhanced by immersing the sample in a deflection medium comprising of an inert liquid FC-72 Fluorinert (3M Company) that has a high refractive index change per unit change in temperature. A fixed wavelength CW transverse laser probe beam, produced using a Qioptiq 670 nm fiber-coupled diode laser with temperature stabilizer for reduced beam pointing noise, was passed through the thermal gradient in front of the sample producing a deflection proportional to the absorbed light at that particular wavelength, which is detected by a differentially amplified quadrant photodiode and a Stanford Research SR830 lock-in amplifier combination. Scanning through different wavelengths gives us the complete absorption spectra.

### Transient absorption

A Ti:Sapphire amplifier system (Spectra Physics Solstice) operating at 1 kHz generated 90-fs pulses was split to given the pump and probe beam arms. The broad band probe beam was generated in a home-built noncollinear optical parametric amplifier. The pulsed excitation was provided by a TOPAS optical parametric amplifier (Light Conversion), to generate narrowband (10 nm full-width at half-maximum) pump pulses of 490 nm for bromide perovskite samples and 650 nm for iodide and iodide–chloride perovskite samples. The transmitted pulses were collected with an InGaAs dual-line array detector (Hamamatsu G11608-512) driven and read out by a custom-built board from Stresing Entwicklungsbüro.

### Transient photoluminescence spectroscopy

Time-resolved photoluminescence measurements were taken with a gated intensified CCD camera system (Andor iStar DH740 CCI-010) connected to a grating spectrometer (Andor SR303i). Excitation was performed with femtosecond laser pulses that were generated in a home-built setup by second harmonic generation in a beta barium borate (BBO) crystal from the fundamental output (pulse energy 1.55 eV, pulse length 80 fs) of a Ti:Sapphire laser system (Spectra Physics Solstice). The laser pulses had an energy of 3.1 eV. Temporal resolution of the PL emission was obtained by measuring the PL from the sample by stepping the iCCD gate delay for different delays with respect to the excitation. The gate width was 1.5 ns.

### Photoluminescence quantum efficiency measurements

The PLQE of the samples was measured using an integrating sphere method, described elsewhere^42^. A continuous wave 532 nm diode laser (407 nm for bromide perovskite) was used to photo-excite the samples. Emission was measured using an Andor iDus DU490A InGaAs detector. The samples were encapsulated between two glass cover slips before measurements.

For pulsed PLQE measurements, excitation was performed with femtosecond laser pulses that were generated in a home-built setup by second harmonic generation in a BBO crystal from the fundamental output (pulse energy 1.55 eV, pulse length 80 fs) of a Ti:Sapphire laser system (Spectra Physics Solstice). PL was recorded with an Andor ICCD (intensified charge-coupled device) after calibration with a calibration lamp.

### Transient PL measurements with TCSPC

The sample was excited with a pulsed supercontinuum laser (Fianum Whitelase SC-400-4, 6 ps pulse lengths) at 0.5 MHz (0.2 MHz for mixed halide samples) repetition rate. The pump wavelength was selected to 490 nm (full-width at half-maximum 10 nm) with dielectric filters (Thorlabs). Pump scatter light from the laser excitation within the photoluminescence path to the detector was filtered-out using an absorptive long-pass filter with a 515 nm edge (Thorlabs). The detection wavelength was selected using dielectric filters (Thorlabs) in front of the detector (>665 nm for MAPbI3 and mixed halide, 514±10 nm for MAPbBr3). The photoluminescence was focused and detected by a single-photon avalanche photodiode based on Si (MPD-PDM-PDF) with an instrument response of circa 300 ps.

### Data availability

The experimental data that support the findings of this study are available in the University of Cambridge Repository (https://doi.org/10.17863/CAM.6537).

## Additional information

**How to cite this article:** Richter, J. M. *et al*. Enhancing photoluminescence yields in lead halide perovskites by photon recycling and light out-coupling. *Nat. Commun.* **7,** 13941 doi: 10.1038/ncomms13941 (2016).

**Publisher's note:** Springer Nature remains neutral with regard to jurisdictional claims in published maps and institutional affiliations.

## Supplementary Material

Supplementary Information Supplementary Figures 1-8, Supplementary Notes 1-2 and Supplementary References

## Figures and Tables

**Table 1:** Recombination constants of MAPbX_3_ planar films(X=I, Br, I_1*−x*_Cl_*x*_).

	**MAPbI**_**3**_	**MAPbI**_**3−*****x***_**Cl**_***x***_	**MAPbBr**_**3**_
*a* (s^−1^) from TA	5e6	≤5e5	2.5e6
*b* (cm^3^ s^−1^)	8.1e−11	7.9e−11	7.0e−11
*c* (cm^6^ s^−1^)	1.1e−28	1.8e−28	6e−29
*a* (s^−1^) from TPL	4.2e6	5.9e5	3.0e6
2·*τ*_PL_ (ns)	230	1,700	330

Recombination constants were extracted from the recombination rates measured with TA (Fig. 2). For comparison, the monomolecular recombination constants were also extracted from low-fluence transient PL measurements.

**Table 2:** Bimolecular recombination constants for planar films of halide perovskites.

**Bimolecular constants in cm**^**3**^ **s**^**−1**^		**MAPbI**_**3**_	**MAPbI**_**3−*****x***_**Cl**_***x***_	**MAPbBr**_**3**_
*b*=*b*_non_+*b*_rad_+*b*_rad,ext_	(from TA)	8.1e−11	7.9e−11	7.0e−11
*b*_non_	(from PLQE)	7.2e−11	5.6e−11	5.4e−11
*b*_rad,ext_	(from PLQE)	0.9e−11	2.3e−11	1.6e−11
*b*_rad,int_=*b*_rad,ext_/*η*_esc_	(calc.)	7.1e−11	18.1e−11	10.1e−11

Internal and external bimolecular recombination constants derived from the measured recombination rates in Fig. 2 and the measured external PLQEs.
