# Supplementary material for: Enhancing photoluminescence yields in lead halide perovskites by photon recycling and light out-coupling
Source: Nat Commun. 2016 Dec 23;7:13941. doi: 10.1038/ncomms13941 (PMC5196482; doi:10.1038/ncomms13941)
Supplement: Supplementary Information — Supplementary Figures 1-8, Supplementary Notes 1-2 and Supplementary References [file ncomms13941-s1.pdf]

Supplementary Information

Supplementary Note 1: Estimate of carrier density

The initial carrier density n after pulsed excitation was estimated using the absorption of the films a, the FWHM of the Gaussian beam as diameter d, the laser power P, the laser repetition rate f, the photon energy $E_{\mathrm{ph}} = \hbar\omega$ and the film thickness $t = 200$ nm. We estimate it to be

$$n_0 = \frac{a \cdot P}{E_{\mathrm{ph}} \cdot f} \cdot \frac{1}{\pi (d/2)^2 \cdot t} \tag{1}$$

Supplementary Note 2: Escape probability for a planar film

The probability for a photon to be transmitted through an interface between two media with refractive indices n_1 and n_2 can be estimated as

$$\eta_{\mathrm{trans}} = \frac{\Omega_{\mathrm{esc}}}{4\pi} T \approx \frac{n_2^3}{n_1 (n_1 + n_2)^2} \tag{2}$$

where Ω_{esc} is the escape solid angle and T is the transmittance [1]. By considering both interfaces perovskite-glass and perovskite-air with refractive indices of 1, 1.5 and 2.7 for air, glass and iodide perovskite respectively, we estimate the transmission probability to be 7.0% for the perovskite-glass interface and 2.7% for the perovskite-air interface. Similar results are obtained by using the expression [2]

$$\eta_{\mathrm{trans}} = \frac{1}{4n^2} \tag{3}$$

Our films had an optical density of ca. 0.05 at the PL emission wavelength. Before reaching an interface, photons will on average have travelled through an optical density of 0.025. If photons are emitted towards the perovskite-glass interface, they will be outcoupled with a probability of 7%. If photons are emitted towards the perovskite-air interface, they will be outcoupled at that interface with 2.7% probability and after reflection at the perovskite-glass interface with (7%-2.7%) probability in the case their emission angle lies in between the critical angles of the two interfaces. We therefore estimate the total escape probability for iodide and iodide-chloride perovskite to be

$$\eta_{\mathrm{esc}} = 10^{-0.025} \cdot \left(7\% + 2.7\% + 10^{-0.05} \cdot (7\% - 2.7\%)\right) = 12.7\% \quad (4)$$

Here, we assume that photons entering the glass will not get back into the perovskite film but have escaped. This is not fully accurate as a certain fraction of these photons will re-enter the perovskite film. However, since the glass is 200µm thick, they will travel long distances in the glass substrate and might re-enter the perovskite film outside the illumination spot. We are therefore slightly overestimating the escape probability here and thus underestimating the calculated internal PLQE in the main paper. The internal PLQE in the main paper is therefore a conservative estimate. For bromide perovskite, the refractive index is 2.4 at the bandedge [3] giving transmission probabilities of 9.2% for the perovskite-glass interface and 3.6% for the perovskite-air interface. With an optical density of 0.6, this gives an escape probability of $\eta_{\mathrm{esc}} = 15.9\%$.

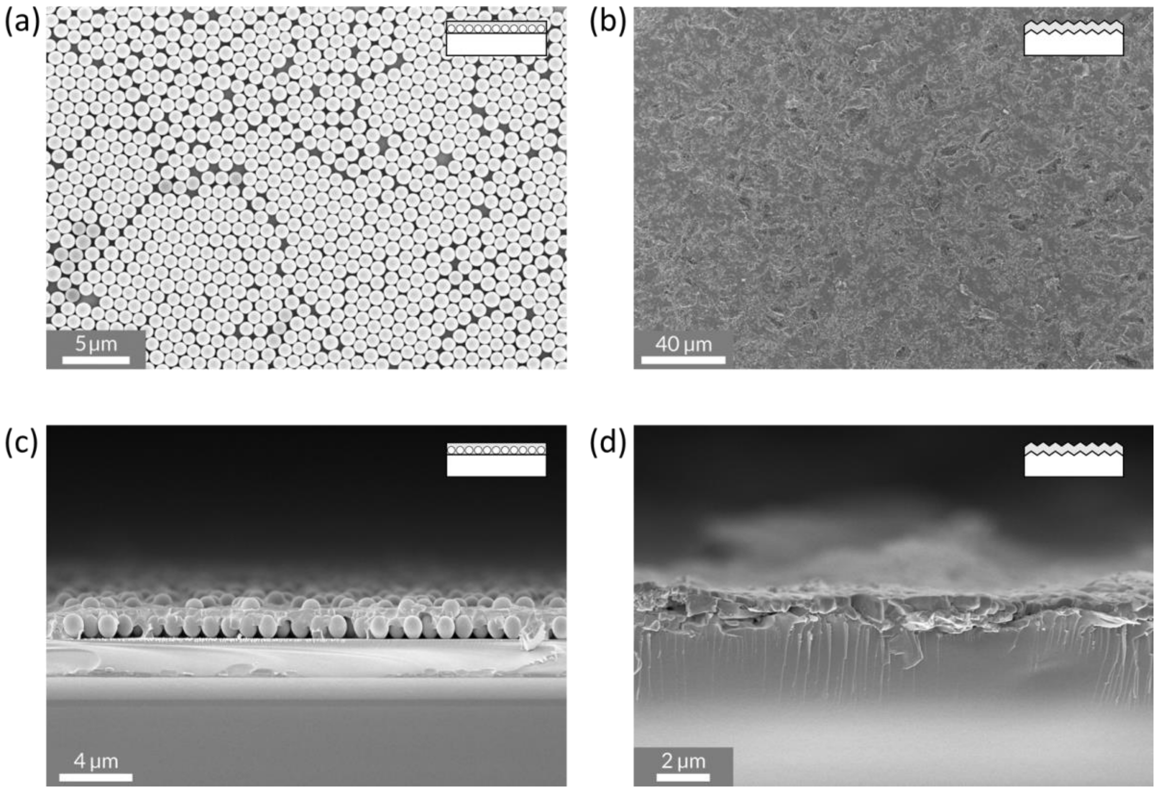

Supplementary Figure 1: Substrate characterisation with SEM. SEM images of (a) the glass substrate with silica beads deposited on it, (b) the rough glass substrate, (c) the silica beads substrate with perovskite deposited on top of the beads layer and (d) roughened substrate with perovskite deposited on it. Scanning electron microscopy images were recorded using a Leo Gemini 1530 VP SEM using an acceleration voltage of V=3kV. (To reduce charging effects of the insulating SiO2 samples, a very thin (<10nm) layer of Au/Pd alloy was sputtered prior to imaging.)

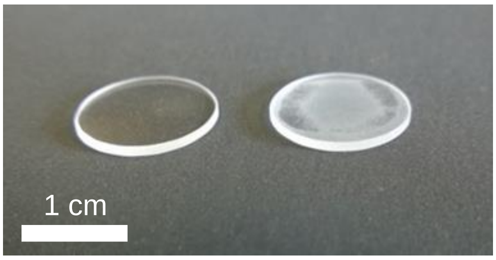

Supplementary Figure 2: Roughening the glass substrate. Photograph of flat substrate (left) and rough substrate (right) after roughening with sand paper.

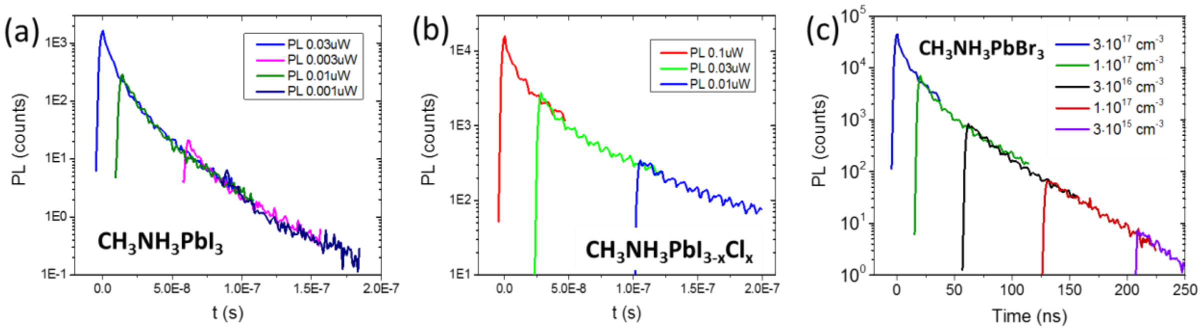

Supplementary Figure 3: PL kinetics are 'history-independent'. Fluence-dependent PL kinetics for iodide, iodide-chloride and bromide perovskite. Time zero of the different fluence measurements was shifted to match the decay of the next higher fluence. We observe that the decays for different fluences match each other very well.

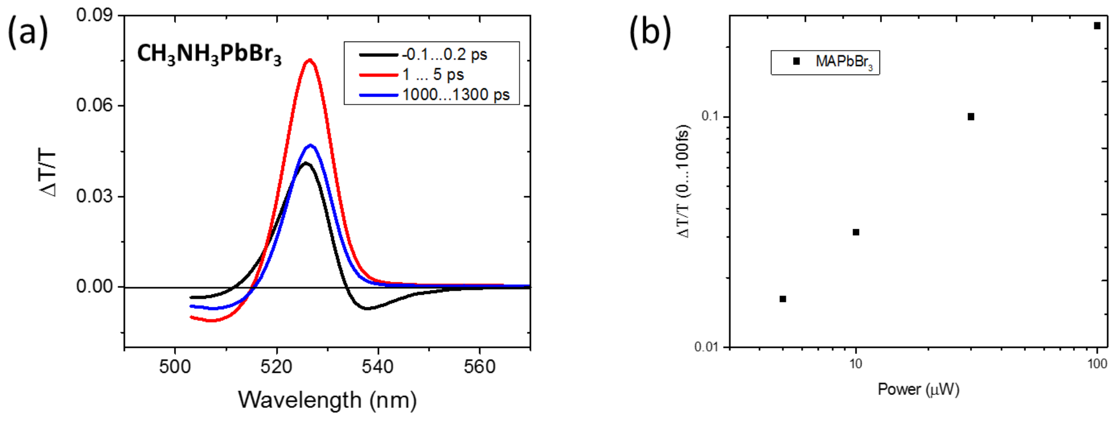

Supplementary Figure 4: Transient absorption spectroscopy. (a) Typical TA spectra for bromide perovskite for different time delays. The strong positive feature at 527nm is the ground-state bleach and was used to track the carrier density. (b) Initial TA bleach signal plotted over pump power. We observe that the TA signal scales linearly in the pump power and thus in the excitation density confirming that it is a good measure for the carrier density and that the measurements were performed in the linear absorption regime.

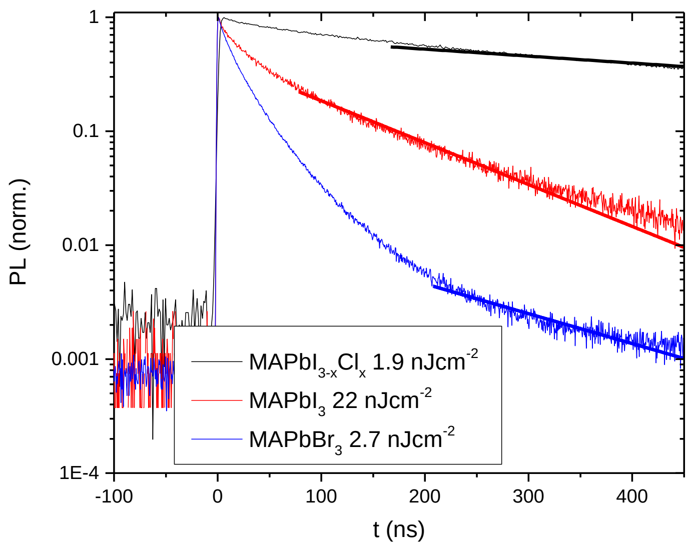

Supplementary Figure 5: Transient PL kinetics of perovskites at low fluences. Low-fluence TPL measurements with exponential fit in the tail with decay times of 850 ns, 115 ns and 165 ns for iodide-chloride, iodide and bromide perovskite, respectively.

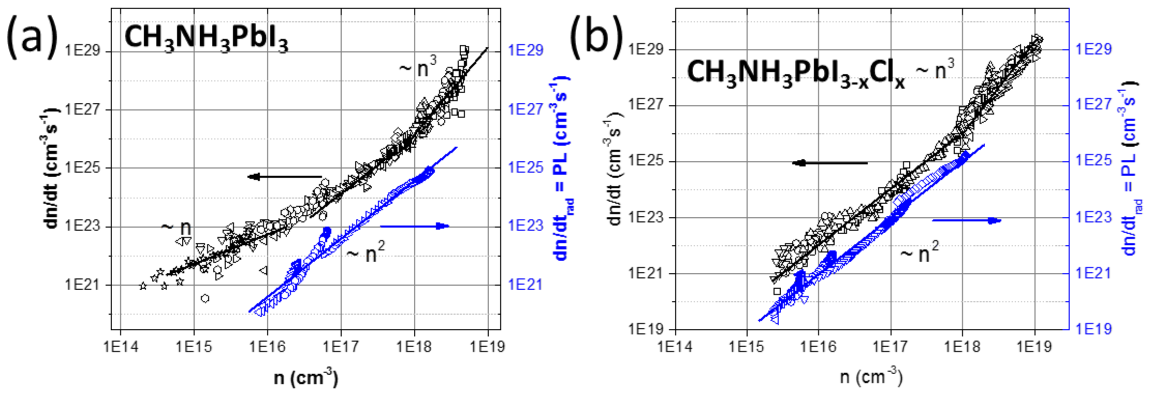

Supplementary Figure 6: Radiative and total recombination rates. Total (black) and radiative (blue) recombination rate dependence on charge carrier density n for (a) iodide and (b) iodide-chloride perovskite. The radiative recombination rates were derived by plotting the transient PL signal over the TA signal at the same time delay.

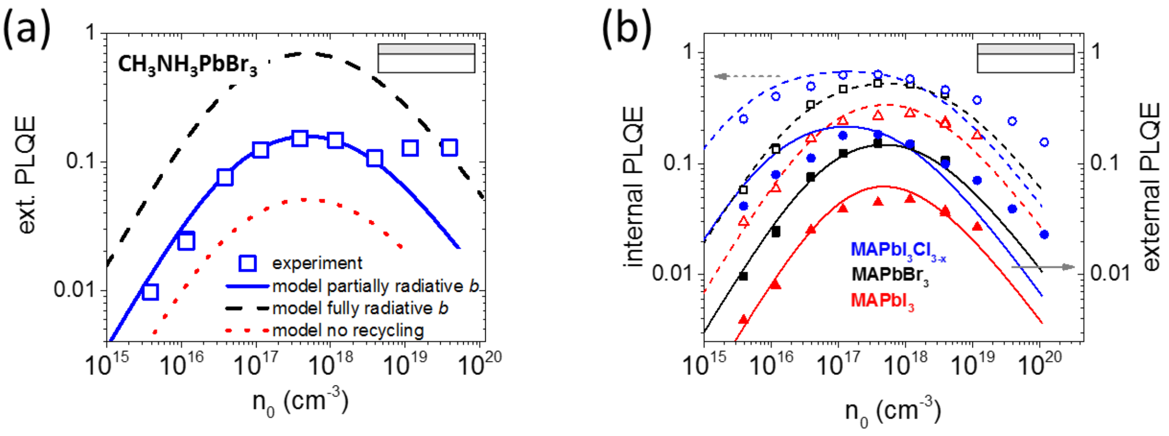

Supplementary Figure 7: Fluence-dependent PLQEs. (a) Measured external PLQE for bromide perovskite (outlined squares) in comparison with different predictions from the rate equation assuming no recycling (red dotted line), recycling and a fully radiative bimolecular constant (black dashed line) and recycling but only a partially radiative bimolecular constant (blue solid line). Above 10^{19} cm^{-3}, amplified spontaneous emission enhances the PLQE leading to a deviation from the rate equation model. (b) Measured external PLQE for iodide-chloride (solid circles), bromide (solid squares) and iodide (solid triangles) perovskites under pulsed excitation and internal PLQE (outlined symbols) calculated from the external ones. The lines represent the model from the rate equation discussed in the main text.

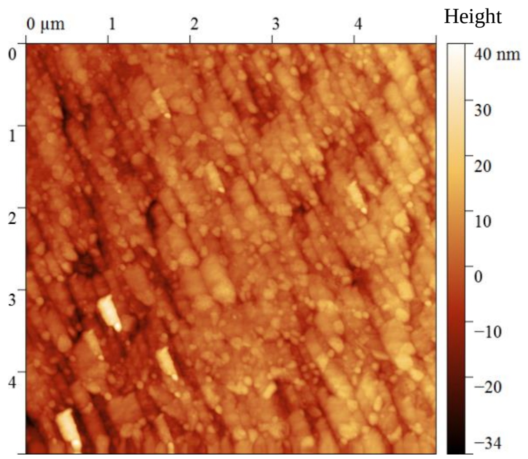

Supplementary Figure 8: Atomic force microscopy. Map of iodide perovskite film surface measured by atomic force microscopy. The film shows a surface roughness of 5.6 nm.

Supplementary References

1. Liu, J. *Photonic Devices*. (Cambridge University Press, 2005).
2. Schnitzer, I., Yablonovitch, E., Caneau, C. & Gmitter, T. J. Ultrahigh spontaneous emission quantum efficiency, 99.7% internally and 72% externally, from AlGaAs/GaAs/AlGaAs double heterostructures. *Appl. Phys. Lett.* **62,** 131–133 (1993).
3. Park, J.-S. *et al.* Electronic Structure and Optical Properties of α-$CH_3NH_3PbBr_3$ Perovskite Single Crystal. *J. Phys. Chem. Lett.* **6,** 4304–4308 (2015).
